# Beyond adoption: The persistence of conservation and climate-smart agricultural practices in the United States

**DOI:** 10.1073/pnas.2518373122

**Published:** 2025-10-21

**Authors:** Paul J. Ferraro, Maria Bowman, Hannah E. Correia, Jing Gao, Kelsey R. Larson, Kent D. Messer, Laura A. Paul, Bryan Pratt, Linda S. Prokopy

**Affiliations:** ^a^Department of Environmental Health and Engineering, Johns Hopkins University, Baltimore, MD 21218; ^b^Carey Business School, Johns Hopkins University, Baltimore, MD 21202; ^c^United States Department of Agriculture, Economic Research Service, Washington, DC 20024; ^d^Department of Biology, University of North Dakota, Grand Forks, ND 58202; ^e^Department of Geography and Spatial Sciences & Data Science Institute, University of Delaware, Newark, DE 19716; ^f^Department of Agricultural Economics and Economics, Montana State University, Bozeman, MT 59717; ^g^Department of Applied Economics and Statistics, University of Delaware, Newark, DE 19716; ^h^College of Agriculture and Life Sciences, University of Vermont, Burlington, VT 05405

**Keywords:** best management practices, disadoption, maintenance, discontinuance, soil health

## Abstract

Achieving sustainability goals requires that humans change their behavior not just once but persistently. Yet despite decades of research on the adoption of conservation and climate-smart agricultural practices, little is known about the extent to which these practices persist over time. One key reason is the lack of longitudinal, field-level data. Using ground-verified, longitudinal data on cover cropping across thousands of farm parcels in Indiana (USA), we find that persistence is low and contrasts sharply with the predictions made by Indiana conservation experts. We also find low persistence in a new national dataset of self-reported cover cropping by farm operators. The potential for low behavioral persistence in sustainable agricultural practices raises essential questions about the design of conservation programs and the modeling and valuation of ecosystem services.

A vast scientific literature characterizes the spatial and temporal diffusion of conservation and climate-smart agricultural practices and the determinants of their adoption (1). Inspired by this literature, federal, state, and nongovernmental actors in the United States use financial and technical assistance to encourage agricultural producers to adopt these practices. Most of this assistance is delivered through short-term (1 to 3 y) and medium-term (10 y) financial contracts, with an increasing share directed toward short-term contracts for “working lands” practices rather than land retirement (over $3 billion in FY2024 alone).^*^

However, for conservation and climate-smart practices to yield their anticipated benefits, they need not only to be adopted but also sustained. Yet, the degree to which these practices persist after adoption and the factors that influence their persistence are poorly understood. A recent review of 35 y of research on the adoption of these practices reported that “[t]here is…little to no focus on adoption over time, a phenomenon that is referred to as maintenance and persistence” (1). Another recent review of agri-environmental financial assistance programs in the United States and Canada reported that their “review of the literature found limited research on whether farmers continue [the practices] after payment ends” (2).

In contrast to the vast literature on practice adoption in the United States, only seven empirical studies have reported on the persistence of working lands practices, of which one reported on behavioral intentions rather than actual behaviors (3). The other six studies came to conflicting conclusions about the degree to which practices persist after adoption (4–9). The differences in conclusions stem from substantial differences in how the authors define “persistence,” as well as differences in data units (operators or counties), populations, and practices analyzed, sample sizes (usually small), and analytical methodologies. None of these studies used ground-verified, longitudinal data at the field level, where the environmental and economic benefits are generated. Thus, our scientific understanding of persistence is rudimentary (10).

## Cover Crop Persistence Is Much Lower than Experts Expect.

To illustrate the potential magnitude of the gap between what persistence may look like on the ground and what experts assume about persistence, we present results from an expert survey and ground-verified, longitudinal data on cover cropping practices.

Cover crops, which cover the soil when fields would otherwise be bare, may benefit adopters and the public by minimizing soil and nutrient losses, increasing soil nutrient content (organic material), and reducing the need for herbicides (11). Despite these potential benefits, cover crops are rare: Fewer than 10% of fields have cover crops annually (12), perhaps reflecting the wide spatial variation in the benefits of cover crops (13). Thus, the US government and its state and nongovernmental partners have prioritized efforts to encourage cover crops on more fields, including the obligation of over $1.49 billion in financial assistance for cover crop use between 2014 and 2024, more funds than for any other working lands practice.^†^ Yet, like many conservation and climate-smart agricultural practices, cover crops require sustained attention and investment to deliver their benefits to producers and the public (14).

To document patterns of field-level persistence in cover cropping and compare them to expert opinions, we use data from a unique state-wide, longitudinal survey of cover cropping. In the survey, staff from the Indiana State Department of Agriculture (ISDA) drive along a fixed route and take observations of fields on the right and left sides of the road every one-half mile. We also conducted an online survey of over 100 Indiana conservation agriculture experts. Respondents were told that the ISDA field survey reported cover crops on 8% of fields in 2014. They were then asked to predict the use of cover crops on these same fields from 2015 to 2019. During the 2014 to 2019 period, Indiana was the second largest recipient of cover crop support from the Environmental Quality Incentives Program (EQIP).^‡^

**Fig. 1.**
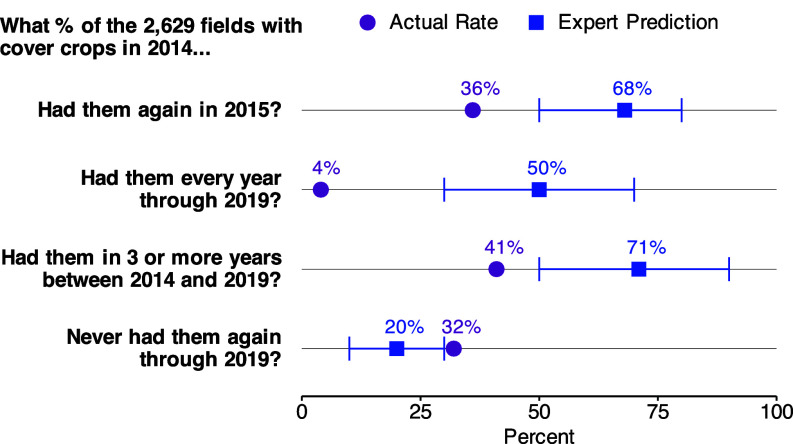
Expert predictions and actual rates of field-level cover crop persistence in Indiana (2014 to 2019). Conservation experts overestimate the persistence of cover crops established in 2014 (N = 110). Red circles represent actual cover crop use in longitudinal, field-level data. Blue squares are median expert predictions of cover crop use in the same fields, and blue bars represent the interquartile range between the 25th percentile and the 75th percentile prediction.

In contrast to the experts’ predictions, the actual persistence rates were low (Fig. 1). While the median prediction was that 71% of fields with cover crops in 2014 would have them in at least three of the six years (2014 to 2019), the field data imply that fewer than half (41%) had cover crops for that many years. This overestimation of persistence arises, in part, because the median respondent believed that 50% of fields would have cover crops every year, whereas the real rate was only 4%. These low rates of persistence may be a problem for achieving conservation goals given that nearly two-thirds of these experts also reported that a “good” or “desirable” outcome would occur only when a field had cover crops on it for at least four out of six years.

Similar persistence patterns are observed in self-reported cover cropping on the United States Department of Agriculture (USDA) Farm Service Agency (FSA) Report of Acreage Form 578, which most US producers fill out each year. For fields with cover crops reported in the first year of the six-year panel (2014 to 2019), only 35% reported cover crops for three or more years, compared to 41% in the ISDA data. Both data sources yield similar patterns of practice cessation: In the ISDA data, 32% of fields with cover crops in 2014 did not have them in any of the next five years, whereas the value is 29% in the national FSA data.

## Implications for Science and Policy.

If these persistence patterns are common across other practices and locations, they have important implications for science and policy.

First, a lack of persistence may shift the focus of financial and technical assistance from new adopters to producers who have previously used a practice. Most current USDA assistance is aimed at new adopters, with EQIP offering contracts only for new practices and the Conservation Stewardship Program encouraging farmers to expand those practices to fields where they have not yet been used. These requirements are motivated by the belief that only newly adopting farmers and fields will provide “additionality”—i.e., changes from what would have occurred without assistance. That expectation implicitly assumes that a producer who adopts a practice will continue it and thus can provide no additionality. If that assumption is wrong, producers who have used practices in the past may offer high levels of additionality. Past adopters may also be less expensive to draw into a program than never-adopters, allowing a program’s funding to reach further.

**Fig. 2.**
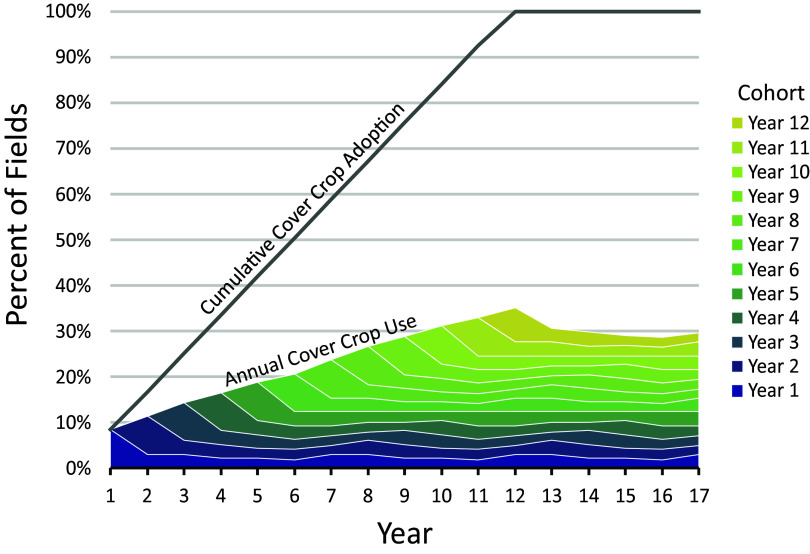
A thought experiment on the limits to aggregate cover crop use. Even if farmers adopt cover crops on 100% of fields, fewer than 30% of fields will have cover crops on them annually in the long term if the patterns of persistence match the patterns observed in Indiana. We assume that cover crops are tried for the first time each year on 8% of all fields until the practice has been tried on 100% of fields after 12 y. The gray line shows the cumulative percentage of fields where cover crops have been adopted, which would be equivalent to the percentage of fields with cover crops only if one were to assume the practice persists on all fields (“Cumulative Cover Crop Adoption”). The colored wedges reflect each annual cohort of newly adopting fields, each of which follows the same six-year pattern of field-level persistence observed in ISDA’s cover crop survey. We assume this pattern repeats forever, which yields a wave-like pattern. The maximum height of the colored wedges shows the percentage of all fields with cover crops on them each year (“Annual Cover Crop Use”).

Second, a lack of persistence means that policy goals for practice uptake may be unexpectedly difficult or impossible to achieve without expanding incentives to previous adopters. Consider, for example, if we assumed that the cover crop persistence patterns among Indiana 2014 adopters were generalizable to the Indiana fields that had not yet adopted. Even if conservation programs could induce producers to try cover crops on every field in Indiana, in the absence of financial incentives for persistence, fewer than 30% of fields would have cover crops in any year because of the low field-level rates of persistence (Fig. 2). To understand whether this pattern can be extrapolated would require knowing the reasons why practices do not persist.

Third, a lack of persistence has implications for the modeling of ecosystem services generated by these practices, as well as economic valuation of those services for use in cost–benefit analyses of agri-environmental programs. Current approaches to modeling and valuation implicitly assume perfect persistence (14). If some practices are subsequently disadopted or only used intermittently, these models will overestimate the benefits to producers and the public.

## Eliminating the Persistence Blind Spot.

Knowing whether these scientific and policy implications are broadly applicable in the United States and globally requires that scientists and practitioners collaborate to eliminate our serious blind spot around persistence. Countries need national-level assessments of the persistence of key conservation and climate-smart practices: nutrient management, edge-of-field conservation, cover crops, rotational grazing, grass waterways, and conservation tillage. Moreover, the research on practice adoption finds that variation is the rule, with few constant predictors across regions and practices (2). Understanding persistence may similarly require detailed studies across a range of settings. To further this goal in the United States, USDA could collect data from financial assistance recipients after the assistance ends, which would help it understand whether recipients continue to use the practice on the same or neighboring fields. USDA could also integrate investigations of persistence into its conservation and climate-smart agriculture programs. For example, USDA and its partners can experiment with renewal contracts for past adopters and contrast the persistence and additionality induced from these contracts with the effects of contracts of varying lengths for new adopters. Only with data and evidence on persistence can we maximize the impact of conservation and climate-smart investments.

## Materials and Methods

Field observations of cover crops in Indiana were from ISDA’s conservation transect survey (also known as a “windshield survey”). FSA data came from the USDA Crop Acreage Reporting Database (CARD). In CARD, cover crops are identified by the stated intended use of a planting. Expert survey respondents were recruited from ISDA, Indiana Soil and Water Conservation Districts, and Purdue University. Human subjects approval was obtained from Johns Hopkins University (HIRB00015270), and informed consent was obtained from all respondents. More details about the data can be found in the *SI Appendix*.

## Supplementary Material

Appendix 01 (PDF)

## Data Availability

Files are available online (15) with the expert survey, respondent data, and description of how the ISDA field data were processed. Access to the ISDA data (https://www.in.gov/isda/divisions/soil-conservation/conservation-transect/) is managed by Samuel Stroebel (SStroebel@isda.IN.gov). FSA data are not publicly available (for more information about access, contact B.P or, if he is unavailable, contact the FPAC/FSA FOIA office directly SM.FP.FOIA@usda.gov).

## Footnotes

^*^Sources: https://www.ers.usda.gov/topics/natural-resources-environment/conservation-programs and https://www.congress.gov/crs-product/IF12024.

^†^Source: https://www.farmers.gov/data/financial-assistance-download. The cited dollar value is for practice code 340. Additional financial assistance for cover crops is also offered under other practice codes.

^‡^Source: https://www.farmers.gov/data/financial-assistance-practices.
