# Supplementary material for: Beyond adoption: The persistence of conservation and climate-smart agricultural practices in the United States
Source: Proc Natl Acad Sci U S A. 2025 Oct 21;122(43):e2518373122. doi: 10.1073/pnas.2518373122 (PMC12582299; doi:10.1073/pnas.2518373122)
Supplement: Supplementary file 1 — Appendix 01 (PDF) [file pnas.2518373122.sapp.pdf]

Supplementary Information for

Beyond Adoption: The persistence of conservation and climate-smart agricultural practices in the United States

Ferraro, Paul J, Maria Bowman, Hannah Correia, Jing Gao, Kelsey R. Larson, Kent D Messer, Laura A. Paul, Bryan Pratt, Linda S Prokopy

Corresponding author: pferraro@jhu.edu

The PDF file includes:

Extended Materials and Methods

Extended Materials and Methods

Annual Cover Crop Survey: Field-level observations of cover crops from 2014-2019 for Indiana were obtained from the conservation transect "windshield survey" conducted annually by staff of the Indiana State Department of Agriculture (ISDA) and supported by the USDA's Natural Resources Conservation Service. In 2021, we requested transect data from 2014 to 2019 to avoid data interpretation and survey execution challenges during the 2020 COVID-19 pandemic. Over the 2014-2019 period, Indiana had annual cover crop use rates that were close to the national average (*1, 2*). ISDA's annual cover crop survey relied on a data collection methodology that was implemented consistently from 2014 through 2019 in every county that had agricultural land (*3*). The annual survey was conducted in each county in autumn. ISDA staff drove along a fixed route and took windshield observations of fields on the right and left sides of the road every one-half mile (see image below, which is part of the survey of conservation experts). The routes were selected to be dispersed evenly throughout the county while also ensuring all regions with heavy agriculture were represented (following recommendations by (*4*)). The windshield observations consisted of crops during the previous season, tillage type, and cover crop status for each field. For each windshield observation, a cover crop was recorded as one or more of the following categories: rye grass, Brassica, cereal rye, winter grains, legumes, oats, wheat, barley, summer mix, plus mix, or none. We defined cover crop presence on a field to be the presence of at least one of the cover crop categories listed, excluding "none". For our analysis, we removed fields that were observed in 2014 but disappear from the data set before 2019. Such attrition was almost entirely a result of a field moving out of agriculture into another land use, and thus into a land cover type that could not experience cover cropping. In other

words, were we to include attritors in our analysis, the persistence rates would be even lower than what we report in the main text.

FSA-578 Data: Completing an FSA-578 form is required if a producer wishes to be eligible for any USDA program. Thus, respondents typically comprise most of the US producers and the country's cropped acreage (5). The responses on this form go into the USDA Crop Acreage Reporting Database (CARD). CARD responses are recorded annually at the level of the Common Land Unit (CLU), which were assigned by researchers at USDA's Economic Research Service (ERS) to "fields." ERS-defined fields are CLUs or parts of CLUs, which are the largest contiguous area affiliated with a common set of CLUs during the period 2013-2019. This processing allows us to assess changes in cropping over time at a field unit that can be constructed to be consistent over time. To align the CARD data analysis with the ISDA windshield survey data analysis, we use the CARD data from 2014 – 2019. Cover crops can be identified in CARD through the stated intended use of a planting. Reporting requirements changed during the 2013 to 2019 period, and thus we use two measures of cover cropping. The measure reported in the main text uses older reporting guidance and classifies a field as having cover crops if the respondent reports the code "CO" (Cover Only) or reports "FG" (Planting for Forage), "GM" (Planting Plowed in for Manure), "GZ" (Planting to be Grazed), or "LS" (Planting Left Standing) while also reporting "GR" (Planting for Grain) or "SG" (Planting for Silage). The second measure uses the most recent reporting guidance and classifies a field as having cover crops if the respondent reports the code "CO" (Cover Only). The first measure, which was used to construct the values reported in the main text, is more flattering to field-level persistence (i.e., compared to the second measure, the first measure provides an upper bound on persistence rates). Because reporting cover crops is not mandatory, both measures may

underreport cover crops. Note that only co-authors B. Pratt (ERS employee), K. Larson (Voluntary Service Agreement, VSA), and H. Correia (VSA) had access to these data, which are not publicly available.

Conservation Expert Survey: The corresponding author and one of the co-authors conducted a pre-registered online survey. The pre-analysis plan (PAP) described recruitment and the survey instrument. The PAP and the response data can be found on the project's Open Science Framework page (see data and materials availability statement). The survey was conducted in Qualtrics.

Here, we reproduce from the PAP the key recruitment details, the invitation letter, the survey questions, and the consent procedure. We also report response rates.

Recruitment: Using online websites for Indiana State Department of Agriculture (ISDA), Indiana Soil and Water Conservation Districts (SWCDs), and Purdue University and an Excel file provided by the Indiana Association of SWCDs, we created a database of individuals, along with their job titles and their contact information, including email addresses. These people were identified by our team as people who, based on their job title or online biographies, plausibly had knowledge of cover cropping patterns in Indiana. We erred on the side of inclusion. Thus, we included field staff, office directors and administrative coordinators. In 2002, separate email invitations were sent to each individual, and two reminders were sent to non-respondents over a period of one month. To reduce the chance that non-invitees would fill out the survey, the invitations included an invitee-specific link. Respondents who started the survey also verified their invitee email address. We have no evidence to suggest that non-invitees responded to the survey. We invited 390 people, of which 7% were from ISDA, 33% from SWCDs, 31% from USDA, 28% from Purdue, and 1% from another organization ("Other").

Responses: Among the invitees, 141 people clicked on the link to go to the survey, of which 8% were from ISDA, 47% from SWCDs, 16% from USDA, 28% from Purdue, and 1% Other. Among those 141 people, 119 completed all or part of the survey, of which 8% were from ISDA, 47% from SWCDs, 14% from USDA, 30% from Purdue, and 1% Other. Based on our discussions with staff at ISDA, we believe that most non-respondents were not field or technical staff (i.e., they were office administrative staff and leaders) and did not work on cover crop-related activities. If the over-representation of SWCD respondents and under-representation of USDA respondents (compared to their proportions in the invitation list) were to bias the responses, we believe that bias would be towards local field staff who would have been the most familiar with recent state-level patterns of cover cropping. Indeed, we believe it would be unlikely for those who are the least familiar with or interested in cover crops to have taken the time to complete our survey. After the survey closed, an online seminar was held to which all survey invitees were invited and the summary data from the survey were presented, along with the corresponding windshield survey data. For the four questions listed in Fig. 1, 115 respondents answered the first question, 111 answered the second and third questions, 110 answered the fourth question, and 110 answered all four questions. In **Fig. 1**, we restrict the sample to the 110 respondents who answered all four questions. If we use all the data, the values are nearly identical. The median predictions [and 25th and 75th percentiles] for the four questions in Fig. 1 are, in order, 65% [50%, 80%], 50% [30%, 70%], 71% [50%, 90%], and 20% [10%, 30%]. One-hundred and thirteen respondents also responded to the following question: “For the 5-year period after cover crops were used on a field in 2014, what do you think represents a “good” or “desirable” outcome in terms of cover crop persistence over time?” Four percent of respondents chose “Cover crops will be used on the field for all 5 years between 2015 and 2019”

(i.e., 6 out of 6 years); 12% chose, “Cover crops will be used on the field for at least 4 more years between 2015 and 2019” (i.e., 5 out of 6 years); 47% chose, “Cover crops will be used on the field for at least 3 more years between 2015 and 2019”; 19% chose, “Cover crops will be used on the field for at least 2 more years between 2015 and 2019”; 16% chose, “Cover crops will be used on the field for at least 1 more year between 2015 and 2019; 0% chose, “Cover crops do not need to ever be used on the field for the subsequent 5 years”; and 2% chose, “I do not know, or I do not believe that there is a “good” or “desirable” outcome in terms of cover crop persistence over time.” In other words, nearly two-thirds of respondents stated that a good or desirable outcome required cover crop use in four or more years out of the six-year period (2014-2019).

Invitation Letter:

From: Laura Paul noreply@surveys.udel.edu
To: {Recipient}

Subject: Cover crop use over time by Indiana farmers. Short survey - respond soon!

Dear {Recipient First Name},

The USDA-funded Center for Behavioral and Experimental Agri-environmental Research invites you to participate in a short 6-question survey about the use of cover crops over time by Indiana farmers. Your answers to these questions are confidential and your name will not be associated with your answers in our analyses or reports. We greatly appreciate your participation!

Follow this link to the Survey (**link expires in 7 days**):

Take the Survey ${l://SurveyLink?}
Or copy and paste the URL below into your internet browser:
${l://SurveyURL}

The link is for you alone. Please do not distribute to others. If you have any questions about this survey, you may contact Dr. Laura Paul at lpaul@udel.edu.

Thank you,
Laura

--
Laura Paul, PhD
Postdoctoral Researcher, University of Delaware
The Center for Behavioral and Experimental Agri-Environmental Research, a USDA Center of Excellence co-headquartered at the University of Delaware and Johns Hopkins University.

Follow the link to opt out of future emails:
Click here to unsubscribe

Survey Questionnaire (unformatted):

[*Page 1*]

Thank you for participating in our short, 6-question survey about the use of cover crops over time by Indiana farmers. We are studying the persistence of cover crop use at the field level. We greatly appreciate your participation. Your answers to these questions are confidential and your name will not be associated with your answers in our analyses or reports.

Duration: This 6-question survey will take about 3 minutes to complete.

Participation: Taking part in this survey is your decision. You can decide to participate and then change your mind at any point.

Contact Information: If you have any questions about this survey you may contact Dr. Laura Paul at lpaul@udel.edu.

Please click "Next" to continue.

[*Page 2*]

Every year, Indiana Department of Agriculture staff conduct a “windshield survey” of cover crop use in the state. They drive the same, pre-specified route late each fall, and they stop at designated points and look left and right. If they see an agricultural field, they record whether there is a cover crop or not.

See the example route from Adams County below.

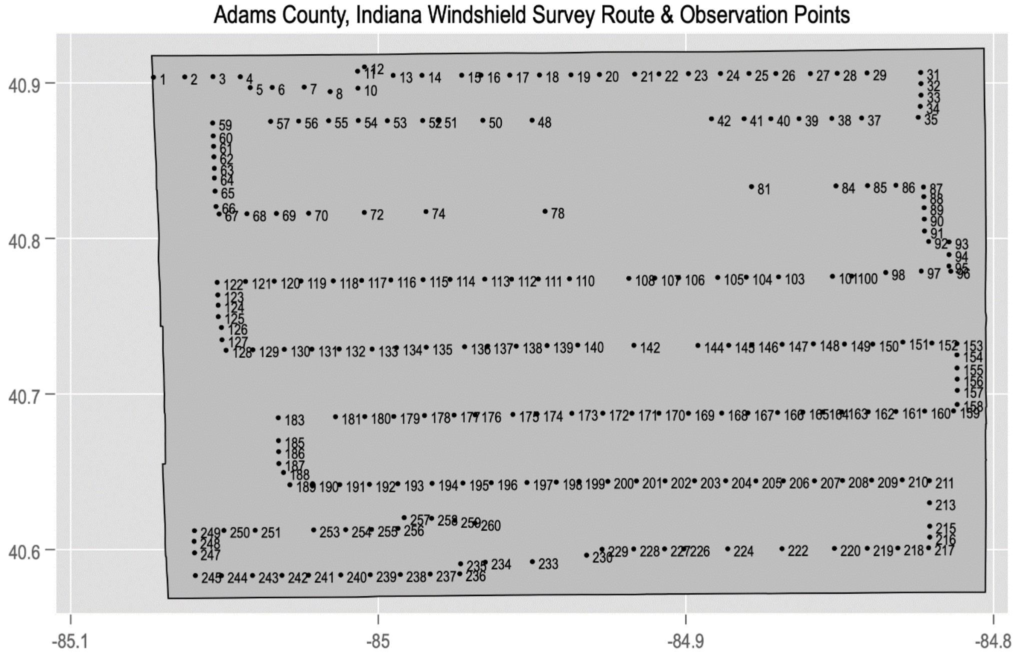

[*Back and Next buttons at bottom of page*]

[*Page 3*]

A field is designated as having a cover crop if the observer sees evidence of one of the following categories of non-weed vegetation:

> Annual Ryegrass, Brassica (radish, turnip, rape), Cereal Rye, Wheat, Barley, Winter Grains (cereal rye, wheat, barley), Legume, Spring Oats, Summer Mix, or a "Complicated Mix" of non-weed species.

This definition of "cover crop" is more expansive than the definition used, for example, in some USDA conservation programs.

[*Back and Next buttons at bottom of page*]

[*Page 4*] [*NOTE TO READER: An error in reading the original data by the project team caused us to report to the survey respondents that there were 2,635 fields in 2014 with cover crops rather than the correct number of 2,629, which is reported in Fig. 1. But because respondents were asked to report their answers as a percentage, we do not believe this minor error affects the interpretation of the results*]

This question asks about your opinion – there is no right or wrong answer.

Between 2014 and 2019, the windshield survey recorded cover crop presence or absence every year on 31,781 fields.

In the 2014 windshield survey, 2,635 fields were reported to have cover crops. In future years, some of those fields will have cover crops again and some will not.

For the 5 year period after cover crops were used on a field in 2014, what do you think represents a “good” or “desirable” outcome in terms of cover crop persistence over time?

- ◯ Cover crops will be used on the field for all 5 years between 2015 and 2019.
- ◯ Cover crops will be used on the field for at least 4 more years between 2015 and 2019.
- ◯ Cover crops will be used on the field for at least 3 more years between 2015 and 2019.
- ◯ Cover crops will be used on the field for at least 2 more years between 2015 and 2019.
- ◯ Cover crops will be used on the field for at least 1 more year between 2015 and 2019.
- ◯ Cover crops do not need to ever be used on the field for the subsequent 5 years.
- ◯ I do not know, or I do not believe that there is a “good” or “desirable” outcome in terms of cover crop persistence over time.

If you would like to elaborate on your answer or make another comment, please use the space below:

[*Back and Next buttons at bottom of page*]

[*Page 5*]

The next five questions have verifiable answers. Within a month of closing the survey, we will provide the answers to every participant who is interested in receiving them.

Between 2014 and 2019, the windshield survey recorded cover crop presence or absence every year on 31,781 fields. In the 2014 survey, cover crops were observed on 2,635 of these 31,781 fields.

Of the 2,635 fields reported to have cover crops in 2014, what percent of them **had cover crops again in 2015**?

Percent of fields that had cover crops again in 2015

[*Slider*] 0 10 20 30 40 50 60 70 80 90 100

[*Back and Next buttons at bottom of page*]

[*Page 6*]

Between 2014 and 2019, the windshield survey recorded cover crop presence or absence every year on 31,781 fields. In the 2014 survey, cover crops were observed on 2,635 of these 31,781 fields.

Of the 2,635 fields reported to have cover crops in 2014, what percent **continued to have cover crops every year from 2015 through 2019**?

Percent of fields that had cover crops every year 2014 through 2019

[*Slider*] 0 10 20 30 40 50 60 70 80 90 100

[*Back and Next buttons at bottom of page*]

[*Page 7*]

Between 2014 and 2019, the windshield survey recorded cover crop presence or absence every year on 31,781 fields. In the 2014 survey, cover crops were observed on 2,635 of these 31,781 fields.

Of the 2,635 fields reported to have cover crops in 2014, what percent had cover crops at least 2 years from 2015 through 2019?

Percent of fields that had cover crops **at least 2 years out of the 6-year period 2015-2019**

[*Slider*] 0 10 20 30 40 50 60 70 80 90 100

[*Back and Next buttons at bottom of page*]

[*Page 8*]

Between 2014 and 2019, the windshield survey recorded cover crop presence or absence every year on 31,781 fields. In the 2014 survey, cover crops were observed on 2,635 of these 31,781 fields.

Of the 2,635 fields reported to have cover crops in 2014, what percent of them **never had cover crops again through 2019**?

Percent of fields that had cover crops at least 3 years out of the 6-year period 2014-2019

[*Slider*] 0 10 20 30 40 50 60 70 80 90 100

[*Back and Next buttons at bottom of page*]

[*Page 9*]

Between 2014 and 2019, the windshield survey recorded cover crop presence or absence every year on 31,781 fields. In the 2014 survey, cover crops were NOT observed on 29,146 of these 31,781 fields.

Of the 29,146 fields reported to NOT have cover crops in 2014, what percent of them HAD cover crops in 2015?

Percent of 29,146 fields that did not have cover crops in 2014 that DID have cover crops in 2015

[*Slider*] 0 10 20 30 40 50 60 70 80 90 100

[*Back and Next buttons at bottom of page*]

[*Page 10*]

Would you like to receive the correct answers to the five questions about the windshield survey?

No

Yes

[*If they click Yes, they can fill in email address in dialog box that appears. Back and Next buttons at bottom of page*]

[*Page 11*]

Would you be willing to answer one more question about the factors that affect the persistence of cover crops over time? If you are, click Yes. Otherwise, click Not Today.

Yes [*Go to screen on page 12*]

Not today. [*Go to screen on page 13*]

[*If they clicked "Yes" above*]

[*Page 12*]

If you change your mind and do not wish to answer this question, you can close your browser at any time. Your other answers have been recorded.

What factors do you believe account for the changes in cover crops presence within fields across time in the Windshield Survey? In other words, what factors lead a field to have cover crops one year but not the next, or vice-versa?

Please express your beliefs in the form of percentages that sum up to 100% (e.g., I believe roughly 20% of the year-to-year variation is due to factor A, 30% due to factor B, etc.)

- 0
  Crop rotation (in other words, the attributes of the crop planted earlier in the year and the crop expected to be planted next year, including decisions to fallow)
- 0
- Change in rainfall in comparison with prior year
- 0
  Change in temperature in comparison with prior year
- 0
  Change in amount or availability of cost-share or incentive payments for cover crops
- 0
  Change in operator's (or landowner's) beliefs about the financial and non-financial rewards from cover crops (independent of changes in cost-share or incentive programs)
- 0
  Change in crop prices
- 0
  Change in operator's cash flow or credit availability
- 0
  Change of landowner or land operator (for example, if the operator in one year is not the same as the operator in another year)
- 0
  Observer error in the Windshield Survey
- 0
  Availability of needed equipment
- 0
  Other
- Total [*automatically computed by system; has to sum to 100%*]

If you would like to elaborate on your answer or make another comment, please use the space below:

[*Back and Next buttons at bottom of page*]

[*Page 13*]

We thank you for your time spent taking this survey.
Your response has been recorded.

References

1. Indiana Conservation Partnership, "Living green covers: 2014-2022" (2023).
2. S. Wallander, D. Smith, M. Bowman, R. Claassen, "Cover Crop Trends, Programs, and Practices in the United States. Economic Information Bulletin Number 222" (Washington D.C., 2021).
3. I. N. R. C. S. & I. C. P. Indiana State Department of Agriculture, "Indiana conservation transect survey 2014-2019" (https://www.in.gov/isda/divisions/soil-conservation/conservation-transect).
4. P. R. Hill, "A roadside survey method for obtaining reliable county- and watershed-level tillage, crop residue, and soil loss data: Procedures for cropland transect surveys" (1996).
5. Office the Chief Economist (OCE), "Update of 2019 FSA Acreage Data and FAQs on USDA Acreage." (Washington, DC., 2019).
